# Hospital and Institutional News

**Published:** 1917-05-12

**Authors:** 


### HOSPITAL AND INSTITUTIONAL NEWS.

### MEDICAL OFFICERS FOR THE R.A.M.C.

During the past week nothing of any note has transpired concerning the position at the War Office regarding the supply of medical officers. According to statements that have appeared in the press, "the United States will send to France 3,000 ambulance cars with 7,700 drivers and doctors. These are additional to the medical units, totalling 1,000 men, to be sent to England." From this rather indefinite announcement it is impossible to judge how many of the 8,700 individuals mentioned will be doctors; but probably not less than 1,000 of them should be so estimated. Such a reinforcement ought to ease the situation in France very materially indeed, though it does not seem to have brought about any slackening so far in the combing-out process at home. Every available doctor of military age is being taken for service, apparently, regardless of what work he does in this country. Only this week we have heard of the death of a child from an acute abdominal condition in one of the best known London hospitals because all the remaining visiting staff happened to be on emergency cases elsewhere, and by the time one of them arrived the case was hopeless.

### MEDICAL PROTECTION.

The annual report of the Council of the London and Counties Medical Protection Society, to be presented at the meeting on May 16, amounts this year to little more than the accounts and balance sheets, whereby a very large saving in paper has been accomplished. As the brief memorandum of the Council very justly points out, the accounts show a most satisfactory financial position and are ample evidence to all whom it may concern of the flourishing state of the Society. Although during the year 1916 the Society added but a few pounds to its balance on the ordinary income and expenditure account, the Insurance Fund and the Reserve Fund show a joint increase of over £3,000 during the year, though against this must be set depreciation of securities amounting to about £1,400, which is doubtless in part merely temporary. The Society now holds, it is stated, £10,000 in British War Securities. Contrary to what might have been expected, with so many members away on war service, the total work of the Society has not diminished; decreases in some respects have been offset by increases in others. At all events, there can be no doubt that this Society is prospering under the extremely able management which it is fortunate in possessing.

### THE LATE DR. J. E. SQUIRE.

The death is announced of Dr. John Edward Squire, C.B., late senior physician to the Mount Vernon Hospital for Diseases of the Chest, at the age of sixty-one. Dr. Squire was a well-known expert in tuberculosis, and became, when the National Insurance Act was launched, the chief adviser to the London Insurance Committee on the sanatorium benefit part of the scheme. He was also at one time physician to the West End Hospital for Diseases of the Nervous System. He was even better known through his long and honourable connection with the Volunteer movement in London. As a young man he served under the Red Cross as senior medical officer (of that organisation) in the Soudan Campaign of 1885, for which he received the Egyptian Medal and the Khedive's Star. He was afterwards for many years an officer in the Volunteer Medical Staff Corps, the predecessors of the Territorial R.A.M.C. After many years of keen and self-sacrificing service he attained the rank of lieutenant-colonel, and on his retirement was made a Companion of the Bath. He was also a Knight of Grace of the Order of St. John of Jerusalem.

### GENERAL DISMAY AT THE SHORTAGE OF DOCTORS.

Owing to the fact that there is every probability of the medical service in Ripon being reduced to two doctors only, the following resolution has been unanimously passed by the Ripon City Council: "In view of the abnormal conditions now obtaining in the city, the large increase of the civilian population, the obviously greater means and area of infection, a reduction of the medical service to only two practitioners would constitute a serious menace. This Council therefore begs to submit a special case for consideration by the Central Medical War Committee, believing that the projected withdrawal of other medical men from civilian practice would be much against the national interest." It was pointed out that the population, including the rural area, is about 14,000 or more. This resolution is a symptom of the general dismay created by the proposal to remove almost the entire profession from civilian work. We trust that it will not pass unheeded.

### INDIA'S RED CROSS GIFT.

The special war fund for the United Provinces of India, inaugurated by Sir James Meston, the Lieutenant-Governor, in the spring of last year, for the provision of the means of transport for the wounded, has had a success that is little short of remarkable. The committee, working in conjunction with the two Red Cross Societies at home, and acting as a rule upon their advice, has expended a sum of £141,700, out of a total of over £200,000 collected, in the purchase of cars, ambulances, and lorries for service on the Western Front and in Mesopotamia. For the former, two convoys of fifty motor ambulances have been provided (one of which was inspected before departure by the King and Queen), together with touring cars to accompany them, and the cost of one year's maintenance. A notable feature has been the equipment of the hospital ship *Nabha*, at a cost of £21,000, of which £15,000 was contributed by the Maharaja of

Nabha, for the transport of wounded from Egypt to England. Ten motor launches have also been equipped for service on Mesopotamian rivers. Such generosity as this forms the best possible answer to the efforts of the German propagandists to foment disloyalty amongst the peoples of our Indian Empire.

### A COURAGEOUS APPEAL.

To ask for £10,000 at the present moment for the necessary if unexciting object of paying off a hospital's overdraft at the bank sounds a bold undertaking, but it is being achieved at Liverpool, thanks to the example set by an anonymous donor of £1,000. He promised this sum if the remainder were subscribed by the end of April, and when that month closed over £8,000 had been received in sums varying from four figures to half a crown. The time limit has now been kindly extended to the end of May, by which time the remaining odd thousand pounds are expected to be obtained without difficulty. The hospital which has accomplished this result is the Royal Southern Hospital, Liverpool, and its successful endeavour is a good instance of what can be done for hospital finances even in the third year of the war.

### LARGE SUMS FOR MIDLAND CHARITIES.

Under the will of Mr. Joseph Taylor, of the Bungalow, Great Barr, Staffs, who died on March 25, several Midland hospitals will receive munificent legacies. Foremost comes the Walsall and District Hospital, which is left the splendid bequest of £20,000. Half that sum is bequeathed to the Walsall Victoria Nursing Institution, while £5,000 goes to the Queen's Hospital, Birmingham. Legacies of £2,000 apiece are provided for a number of charities, amongst which are the Birmingham and Midland Eye Hospital, the Wolverhampton and Midland Counties Eye Infirmary, the Midland Counties Home for Incurables, Leamington, and the Royal Orphanage, Wolverhampton. There are a few personal bequests, after which the residue of the property, the net personalty of which is valued at £83,228, goes to the British and Foreign Bible Society.

### A DOCTOR'S IMPORTANT BEQUEST.

The enthusiasm which the creation of the Welsh Medical School has excited in the Principality is well shown in the will of the late Dr. William Price, of Cardiff, who bequeathed all the residue of his estate, which is estimated at about £20,000, to the medical school of the University College of South Wales and Monmouthshire. The principal of the college is one of his executors and trustees. It is noteworthy that the general public, as well as the medical profession, has rallied to the school, and this solid support, we may be sure, will carry the project through in spite of all the checks which the war or official delays may impose upon it.

### "THE SILVERTOWN COT."

The manufacturers in the neighbourhood of the explosion on the night of January 19, being desirous of recognising the services rendered by the Albert Dock Hospital of the Seamen's Hospital Society, which bore the brunt of treating the sufferers in connection with that catastrophe, have given a sum of £500 to the hospital to name a bed to be called "The Silvertown Cot." A tablet will be placed over the bed bearing the following inscription: "*The Silvertown Cot.* This tablet is erected by manufacturers in this district to commemorate the services rendered at this and other local hospitals and institutions on the evening of the explosion—January 19, 1917."

### THE PRINCE OF WALES' HOSPITAL FOR LIMBLESS SAILORS AND SOLDIERS, CARDIFF.

The Prince of Wales' Hospital, Cardiff, was inspected by Surgeon-General H. Hathaway, C.B., D.D.M.S., of the Western Command, on Thursday, May 3, and on Friday, May 4, a visit was paid to the hospital by King Manoel, who was accompanied by Colonel Robert Jones, C.B., Inspector-General of Military Orthopædics. The ten wards (which will accommodate fifty-three men) present a cheerful appearance, and are quite completed. So also are the dining-hall, kitchens, and limb workshops. The technical trades workshops (covering engineering, electrical, wood-working, and clerical departments) will probably be fitted up during the next few weeks. Pending the completion of the large recreation-room (which will be a feature of the hospital), one of the training workshops will be used for this purpose. This hospital will serve for all men "domiciled in Wales and the Border counties." In view, therefore, of the large prospective number of patients, the executive committee has given careful consideration to the ways and means by which "vacant beds" may be avoided, in order that the efficiency of the hospital may be kept at a high level. It has been arranged (by the hearty cooperation of the staff of the Welsh Metropolitan War Hospital, Whitchurch, Glam.) that, as far as possible, the patients shall be received through the Whitchurch Hospital. The two hospitals are only three miles apart, and will be in daily telephonic communication with each other. It is hoped thus to keep the "Fitting Hospital" (the Prince of Wales' Hospital) fully tenanted, so that the greatest possible number of patients may be treated during the year.

### STAFF PUZZLES IN WARTIME.

The Royal West Sussex Hospital, Chichester, is not the only one which has experienced staff troubles during the war, but it is typical of the times that in the course of the past year the matron, all the sisters, and many nurses and probationers left to take up other work. The new matron, Miss Parsons, had therefore to create a fresh staff on her arrival, and at the present time that is no easy task. The former acting house surgeon is now serving in France, and his successor, a former house surgeon who happened to be home on leave from Australia, is leaving. There have been five applicants for the post: three are not of European nationality, one is a woman doctor, and one is an Englishman. The latter is the hope of the board; but what a record is this of the problem of staffing a hospital in wartime!

### ONE THOUSAND SANATORIUM BEDS IN WALES.

At the recent Council meeting of the Welsh National Memorial Association the general director, Mr. D. W. Evans, stated that there were now 955 beds in Wales compared with thirty-one seven years ago. The Association had decided to maintain 1,000 beds, but in order to do so it is necessary for the contributing Councils to raise their rate in aid from a halfpenny to three-farthings in the pound. An agreement to do so is not unlikely, and if it is made the Association, in spite of criticism, will have an impressive achievement to show for the enthusiasm which started, and the contributions which maintain it.

### HOSPITAL FINANCE.

The problems presented to hospital committees by the ever-rising cost of raw materials and foodstuffs is reflected in several of the annual reports now to hand. At the Morning Field Hospital, Aberdeen, the cost of maintenance per patient is stated to have been £35 9s. 6d., a rise of nearly £3 over the previous year's figures, and, in spite of special payment for forty military cases, there was a deficiency of £217. At the Westmoreland County Hospital the cost per patient per week was £1 5s. 3½d., as compared with £1 3s. 7¼d. in 1915, and this in spite of many generous donations of game, fruit, and vegetables. A notable feature was the public-spirited action of the dairyman who continued to supply milk at 2½d. a quart when the retail price in the town was 4d. The Birmingham Homœopathic Hospital shows a deficit of £595 on the year's working, attributed to the increased cost of maintenance, 29 per cent., representing a cost of 13s. 5d. per patient per week. As there is no endowment fund to draw upon, the position is sufficiently serious. There was a gratifying increase in public subscriptions, which did not, however, counterbalance the increased expenditure. A notable feature in nearly all the reports is the falling off of subscriptions. This is little to be wondered at when one considers the innumerable appeals now being made to the charitable public. It is to be hoped that the needs of the military, however urgent, will not be allowed to eclipse the claims of these indispensable public institutions.

### ST. ANDREW'S HOSPITAL, DOLLIS HILL.

The Lord Mayor of London is appealing broadcast to the public for funds to reduce the building debt of this hospital, now standing at £9,000. He proposes on May 24 to pay an official visit in civic state, by which time he hopes to have sufficient response to his appeal to be able to clear off the debt. It is claimed that since the outbreak of war this hospital has, without relaxing its ordinary work, offered all its available beds for the treatment of wounded soldiers, a claim which, as everyone knows, applies to most of the voluntary hospitals of this country. The Queen of the Belgians is the patroness of this hospital, which was founded in 1913, and has always reserved some of its beds for patients of French-speaking countries, and the patron is Cardinal Bourne. Inasmuch as this hospital is in some degree a sectarian (Roman Catholic) institution, it may be questioned whether a general appeal is tactful.

### LIFE ASSURANCE OF MEDICAL MEN ON WAR SERVICE.

Many of the medical men affected by the decision to establish more hospitals oversea are naturally interested in their position as regards life assurance. At the moment, many offices are not willing to cover any risk arising from active war service abroad, so that it is by no means easy to obtain a suitable policy. The Medical Insurance Agency has been able to make arrangements with a well-known office, under which, the *British Medical Journal* is informed, all risk of foreign service can be insured on payment of an extra premium, or the war risk can be suspended without ultimately invalidating the policy. Under the first plan the extra war premium becomes payable when the doctor leaves the country; but if he should never go within ten miles of the firing zone, at least 50 per cent. of the extra is refunded on his return to this country in good health. Under the second plan policies can be issued under certain tables, without extra rate, in which the risk of death is excluded during active service abroad or within six months thereafter. At the expiry of the period of suspension the risk revives without further medical examination or inquiry, and should death occur during the period, the premiums paid are returned. The first method appears to be the better one, and the feeling that his dependants are in a reasonable measure provided for must be heartening to the medical man setting forth to encounter risks the extent of which cannot be definitely foreseen.

### AN UNSOLVED PROBLEM OF PUBLIC HEALTH.

Last week the Municipal and County Engineers discussed a paper on "The Dustbins of London," and this week the Royal Sanitary Institute debated a paper by Mr. C. Cooper, Borough Surveyor of Wimbledon, on "The Collection and Disposal of Refuse." It is sufficient to listen to discussion and hear so many different views to realise that what on a cursory examination seems a simple matter is really full of perplexity. Mr. Cooper declared that methods of collecting house refuse have not improved in one hundred years, and he showed an old print of a dustman and cart of a century ago—a picture which could pass muster for a present-day illustration. Then dustmen had ladders to enable them to shoot the refuse in the carts. Now they also have ladders. There has been no genius in these hundred years to build "low-level" carts so that dustmen could do without ladders. Even with modern motor vehicles ladders are necessary. The present is not an opportune moment to introduce reforms because of the scarcity of labour, but when peace comes the discussion on this ancient problem might conduce to modern methods being introduced. Meanwhile it is obvious that no two engineers are agreed on the best method of collecting and disposing of house refuse.

### THE UNHEALTHY CINEMA.

The picture palace is admittedly no place for good health, and it is pleasant to know that some authorities are taking steps to render such places less objectionable. At York the City Council is requiring proprietors of such places to sign an undertaking, when applying for licences, to the effect that all windows and other means of ventilation shall be kept open and utilised for at least three hours daily in the morning for the admission of fresh air and diffused daylight or sunlight. Another valuable clause which they must sign is: "If, in the event of a serious epidemic of infectious disease within the municipal area of York, the Health Committee, on the recommendation of the Medical Officer of Health, by notice directs the licensees to exclude from the cinemas for such period as it thinks fit all children under the age of fourteen years, no child under such age shall be permitted to enter or be in the licensed premises at any time during the period named."

### THE MANUFACTURE OF ARTIFICIAL LIMBS.

Pressure is being brought upon the Government by M.P.s and others, who are taking a keen interest in the question of providing disabled sailors and soldiers with artificial limbs, to make the manufacture of such limbs a department of the State's wartime service. It is urged that, if the Government took over this work from the contractors and organised it efficiently, limbs could be more cheaply supplied than at present. In the case of officers the Government makes a grant of £25 where the purchase of an artificial limb is necessary, but not infrequently the patient is called upon to pay anything up to £40. Undoubtedly there is a hardship here which should not exist, but it could be remedied without the setting-up of State workshops for the manufacture of artificial limbs, in the hope that a saving—which is very problematical—would be effected. Up to the end of April no fewer than 6,000 of our soldiers had been furnished with mechanical limbs.

### EPIDEMICS IN ROUMANIA.

The correspondent of the *Times* at Jassy, in a dispatch nearly six weeks old, gives a tragic account of the devastations wrought by typhus and cholera amongst both the military and civil population. Late winter and early spring are, in the Balkans, almost invariably accompanied by local outbreaks of the exanthemata, but from what one can gather, the story of the Serbian epidemic of 1915 is now being repeated on an almost equal scale. Some 25,000 to 30,000 patients were in Bukarest before the entry of the Germans, and at least 40 per cent. of these had to be left behind. Of the remainder, not a few failed to survive the rigours of the journey, and at Jassy itself there was little or no hospital accommodation available. Overcrowding, coupled with a severe winter and great scarcity of provisions, provided all the needful factors for an epidemic, in which many of the doctors and nursing staff succumbed. Its horrors can only be adequately pictured by one who has seen the like in other countries of the Balkan peninsula. It is, nevertheless, difficult to understand why, with our increased knowledge of the causation and prevention of typhus, more vigorous measures have not been taken to stem its course. As it is, the approach of milder weather must be awaited, if, indeed, it has not already arrived, before there can be any hope of the epidemic lessening in severity.

### THE PUERPERAL DEATH-RATE IN ANGLESEY.

A startling fact was mentioned by the medical officer in a report to the Anglesey County Council last week—namely, that the deaths in child-birth are nearly double in Anglesey what they are in London. He complained that unqualified midwives signed maternity claims as "certified midwives," and appealed to the approved societies to co-operate with the County Council in protecting and affording guidance to the wives of working men. Many mothers have found it an impossibility to procure sugar necessary for their bottle-fed babies, so the County Council, on the medical officer's recommendation, has applied to the Food Controller for an allowance of sugar, which it will distribute free in small amounts to poor parents in the county.

### AVERAGE OCCUPIED BEDS: ONE.

There are twenty beds at the Easington and Sedgefield Smallpox Hospital, and since the building of the hospital some years ago there has only been one bed occupied—in fact, there has only been one patient. The year in which the reception of the unique patient took place must have produced amongst institutions a record in bed occupancy which it will be hard to beat. Some discussion took place at a recent meeting of Easington Rural District Council; the main point raised was in regard to the expense involved, and it was argued that it is better to spend money in preventing a disease than to spend more afterwards to counteract it. The incidence of smallpox upon a particular neighbourhood naturally varies from year to year, and it is likely that an outbreak, comparatively large for the district served by the hospital in question, quite justified its establishment. If it were closed, such is the contrariness of things that it might be needed soon afterwards. In any case, the cost of maintenance cannot be large; in respect of medical and nursing service it can be easily arranged that no full-time appointments are made, and the matter of keeping the hospital aired and ready is a small and inexpensive one.

### NON-RESIDENT IN-PATIENTS.

A noteworthy array of military patients has been treated at the Royal Mineral Water Hospital, Bath, during the past year. Out of a total number of 1,107 admitted, no fewer than 762 were soldiers, while of the 341 civilians many were either men recently discharged from the Army or munition workers. Such a proportion is large, but it is fair to add that civilians have not been wholly neglected.

A scheme was tried for the treatment of women who would, under ordinary circumstances, have been admitted as in-patients. For these, advice, drugs, massage, and electrical treatment were given at the hospital, and free baths were provided for them at the Corporation's establishment, but they had to provide their own board and lodging. This scheme seems to have been successful, and 123 women were treated under its provisions. It is a good example of the shifts necessitated by the war.

### HOSPITAL STAFFS AND THE TRIBUNALS.

The appeals of several members of the staff of the Lord Derby War Hospital were lately heard before the Warrington Rural District Military Tribunal. One was a masseur, who was stated to have received official recognition, and for eighteen months to have treated 480 men a month. He had till lately been covered by a military certificate, but the hospital authorities did not proceed with their appeal on his behalf, and the certificate was stated to have been withdrawn under orders from the War Office. When the chairman of the Tribunal asked whether the War Office was not the best judge of the most useful employment of the man, his solicitor replied that in his experience the military mind is not even the best judge of what is good for itself. The military representative denied the man's indispensability. The Tribunal granted exemption till June 1. An upholsterer employed at the hospital received one month's exemption on domestic grounds, and a driver and electric-wireman, also employed at the hospital, was granted exemption for two months for the same reason.

### THE DEATH OF A VETERAN GUARDIAN.

The recent death of Mr. John Feetham, at the age of eighty-four, removes a man whom his friends in Darlington claim to be the oldest Guardian in England. At the age of twenty-five he began to represent the parish of Great Burdon, and he remained a Guardian for sixty-one years, and for no fewer than forty-two years was chairman of his board. In this long period, as he once confessed, he had seen the creation and decay of Highway Boards, School Boards, Rural Sanitary Authorities, and School Attendance Committees, and the great changes which have taken place in the workhouse. He also became chairman of the bench, and his long and useful life was filled with work in a multitude of public offices.

### A DEPUTATION TO LORD RHONDDA.

Representing various organisations interested in maternity and infant welfare, an influential deputation was received by the President of the Local Government Board last Monday. Associated with Lord Rhondda on this occasion were Mr. Hayes Fisher, the Parliamentary Secretary of the Board, and Mr. Monro, the President of the Scottish Local Government Board. The deputation included a number of women and medical men connected with social work coming within the sphere of the subject. Dr. Still, the Chairman of the National Association for the Prevention of Infant Mortality, was the spokesman, and he urged the creation of a Ministry of Health, which should place in the forefront of its programme the care of the health of infants and young children. He attached paramount importance to the health of what he called the "pre-school" age, as statistical returns had proved that there is no period in life up to sixty-five years of age that showed so heavy a death-rate as the first five years of life, and in infancy (the first two years) by far the heaviest mortality was shown. This result is largely due to preventable causes, the chief being faulty feeding. Not only wastage of life is concerned; the marring and maiming of survivors requires equally careful attention. Dr. Still touched upon a variety of apposite social conditions, pointing out that the care of motherhood bears a close relation to the health of the infant. Competency in midwives, suitable industrial conditions, and proper housing are essential. Lord Rhondda said that the question of a Ministry of Health is still *sub judice*, but hinted that if it were created it would not be a new Ministry (there being too many Ministries), and he hoped it would be largely based on the present Local Government Board. Whatever happened, efforts would continue on behalf of maternity and infant welfare, and a Bill would be introduced providing for the feeding of expectant and nursing mothers and to secure pure milk for the children.

### A SCHOOL CLINIC AT LEEDS.

The report of the year's working of this clinic, by Dr. Lee Bolton, gives ample proof that its establishment has been of the utmost benefit to the city as a whole. Much stress is laid upon the undue incidence of sickness, due to improper feeding and national ignorance. "Few mothers nurse their children, or if they do nurse them, do so for too short a time. The consequence is that these children are brought up on proprietary articles of food—many of them are only starch and saccharine, which fail to supply the material to form bone and sturdy muscle." Operative treatment, whether by the family physician, hospitals, or infirmaries, is quite satisfactory; but where directions are given to be followed at home many parents, through ignorance or indifference, fail to follow the instructions given them, causing much unnecessary suffering and absence from school. Of reforms, he advocates the establishment of a "ringworm" school, for the efficient treatment of this malady without loss of attendance, and of open-air schools for weakly and debilitated children, where they would increase their powers of resistance and have a better chance of recuperation after a long illness. During 1916 19,752 examinations were made, as against 17,628 in 1915; and 9,250 children were reported as requiring treatment. There was also, we are glad to learn, a decrease in the number of children showing signs of subnormal nutrition; 13.1 per cent. this year, as compared with 14.6 last year. This improvement he attributes to the better industrial conditions now prevailing. Be that as it may, that one child out of every eight should be allowed to start life crippled by parental neglect or poverty represents a loss of efficiency which in these times the nation would do well to consider.

### WAR WORK AND WOMEN'S HEALTH.

The annual report of the Birmingham Women's Hospital throws an interesting sidelight on the ill-effect of arduous war work upon the physique of women engaged upon it. In 1914 there were 1,494 admissions; in 1915, 1,655; and 1,727 in 1916. There was in consequence an increase of the operations, which numbered 1,594, as compared with 1,503 in the preceding year. The president, Mrs. John Ferney, in moving the adoption of the report, drew attention to the deficit of nearly £3,000, which is entirely due to the increased cost of provisions and other necessaries. The deficit increased by £600 during the year, although special efforts were made to reduce it. Moreover, the increasing calls made upon the hospital, owing to its growing popularity and the larger increase in women's ailments, rendered the supplementing of the usual funds a matter of urgent necessity. It was decided to ask all patients who could afford it for a small donation. The work of the new matron, who entered upon her duties just before the last annual meeting, was spoken of in the highest terms. Under her supervision the nursing reached a very high standard, and the most rigid economy, as shown by the accounts, is a testimony to the satisfactory work she has done.

### A LARGE ESTATE GOING BEGGING.

When Miss Rose Green, of Titchfield, Hants, made her will the cost of maintaining a convalescent home was probably a less expensive matter than it proves to-day. Miss Greene left the Catisfield House Estate as a convalescent home for ladies, and £14,000 Consols for the endowment of the new institution. The £350 a year that the stock represents would not go very far to maintain a home of any importance; even if the Consols have been converted into War Loan Stock, and made to produce a rather larger income, it would not greatly improve the outlook. But the trustees have an alternative scheme under the bequest, which is interesting, if not less perplexing. Within twenty-one years of the death of the testatrix, should they determine such a home to be impracticable, the estate and the sum of Consols may be transferred to the convalescent home attached to any specific hospital the trustees may choose. The best plan, therefore, seems to be for the estate and money to be left to lie fallow, and meantime enterprising secretaries of hospitals requiring convalescent homes, or wishing to strengthen the financial position of existing homes, will cultivate the acquaintance of the executors with assiduity.

### RESIGNATION OF THE MATRON OF WORCESTER GENERAL INFIRMARY.

Miss Mary Herbert, who has been matron of Worcester General Infirmary since 1894, has resigned her appointment, thus severing her official connection with that institution after twenty-three years' devoted service. Trained at St. Thomas's Hospital, where she was subsequently sister, night superintendent, and assistant matron, she brought invaluable experience to aid her in her work at Worcester, which has not been without its difficulties. Miss Herbert has proved herself worthy of the best traditions of St. Thomas's Hospital and the Nightingale School. Her resignation is deeply regretted by the authorities at Worcester, which is but just, seeing that the late matron devotedly laboured with a courage and persistence which did her honour, despite the continuous obstacles to progress which she has had to face during most of the years of her service at the County Hospital there. We hope she may still find congenial work, for a career like hers should fittingly exhibit continued activity on her part throughout life. An account of the presentations to Miss Herbert will be found on p. 120 of the present issue.

### HOSPITAL EXTENSION AT ASHTON-UNDER-LYNE

Funds are coming in satisfactorily towards meeting the cost of the extension scheme in connection with the Ashton-under-Lyne District Infirmary. The proposed new wards will add a further twenty-eight beds to the institution, and additions are also to be made to the nurses' home. The estimated cost of the new buildings as planned is £4,000, and over £3,500 of this amount has now been either subscribed or promised.

### QUININE FOR THE ARMY.

A notice issued by the Army Council on the 2nd of this month announces their intention to take possession of all stocks of quinine, phenacetin, and formaldehyde. Firms holding small stocks of these chemicals or having them actually in transit are allowed to dispose of them as they wish, but with this exception all quantities must be held at the disposal of the War Office. Though doubtless dictated by necessity, such a measure as this, neglecting as it does the needs of the civilian population, must require considerable urgency for its justification. It will be interesting to learn if other drugs are to be commandeered at an early date.

### THIS WEEK'S DRUG MARKET.

With the exception that one or two small holders of quinine appear to have been informed by the Government that they can sell, there have been no developments in the market for the three drugs —quinine, formaldehyde and phenacetin—affected by the Army Council Order alluded to in the last report. On the whole, business is rather quiet, and the upward tendency of prices continues. Cream of tartar is again dearer; the advanced prices of citric and tartaric acids are well maintained. Owing to the further advance in the price of cloves there is likely to be another rise in the quotations for clove oil. Olive oil is extremely scarce. There has been a further sharp advance in the price of sugar of milk. Hypophosphites continue rather scarce, but the demand is not very large at the moment. No English henbane leaf is offered, but a small bale of imported leaf is offered at an extreme figure. A remarkably high price is asked for colchicum corm. In synthetic drugs there have been few changes; the upward tendency in the price of sodium salicylate continues, and hexamine is dearer.

